# Sb^3+^-Doped Rb_2_HfCl_6_ Perovskites as High-Performance Thermally Stable Single-Component Phosphors for White Light-Emitting Diodes

**DOI:** 10.3390/ma18091896

**Published:** 2025-04-22

**Authors:** Yanbiao Li, Yuefeng Gao

**Affiliations:** College of Marine Engineering, Dalian Maritime University, Dalian 116026, China; dmulyb@dlmu.edu.cn

**Keywords:** lead-free perovskite, self-trapped excitons, tunable luminescence, WLED, thermal stability

## Abstract

Stable and efficient inorganic lead-free double perovskites are crucial for high-reliability optoelectronic devices. However, dual-doped perovskite phosphors often suffer from poor color stability due to differences in thermal activation energies and electron–phonon interactions between the doped ions. To address this, single-doped Sb^3+^-incorporated Rb_2_HfCl_6_ perovskite crystals were synthesized via a co-precipitation method. Under UV excitation, Rb_2_HfCl_6_:Sb exhibits broad dual emission bands, attributed to singlet and triplet self-trapped exciton radiative transitions induced by Jahn–Teller distortion in [SbCl_6_]^3−^ octahedra. This dual emission endows the material with high sensitivity to excitation wavelengths, enabling tunable luminescence from cyan to orange-red across 400–800 nm. Utilizing this dual emission, a white LED was fabricated, showcasing a high color rendering index and excellent long-term stability. Remarkably, the material exhibits breakthrough thermal stability, maintaining more than 90% of its emission intensity at 100 °C, while also exhibiting remarkable resistance to humidity and oxygen exposure. Compared to co-doped phosphors, Rb_2_HfCl_6_:Sb offers advantages such as environmental friendliness, simple fabrication, and stable performance, making it an ideal candidate for WLEDs. This study demonstrates notable progress in developing thermally stable and reliable optoelectronic devices.

## 1. Introduction

In contemporary lighting technology, phosphor-converted light-emitting diodes (pc-LEDs) have gained widespread attention across various applications, emerging as the predominant light source owing to their advanced technology and superior photonic characteristics [1,2]. Typically, pc-LEDs are fabricated through the integration of a blue or ultraviolet LED chip with a mixture of red, green, and blue phosphors [3,4]. Nevertheless, several technical challenges persist, including energy loss through inter-phosphor reabsorption, inconsistent degradation rates among different components, and intricate production procedures, all of which contribute to elevated production expenses and diminished light conversion efficiency [5,6]. Furthermore, traditional LED suffers from numerous problems under high-temperature conditions, such as brightness decay, thermal failure, or even burnout, making it difficult to meet the lighting demands in special environments like automotive, outdoor, and industrial lighting, severely restricting the large-scale application of LEDs [7,8]. Recent research has increasingly focused on single-phase luminescent material-based LEDs, driven by their superior color rendering, simplified device configuration, and potential to address the challenge of degradation rates of various components [9,10]. Moreover, single-component LED materials exhibit significant advantages in high-temperature resistance, effectively addressing the performance degradation issues encountered by conventional LED materials in high-temperature environments [11,12,13]. Therefore, the development of single-component LED materials with robust high-temperature resistance is crucial for enhancing the reliability of LEDs and expanding their range of applications.

Lead-free metal halide perovskites have attracted significant interest due to their tunable band gaps, wide color gamut, high absorption efficiency, long carrier lifetime, and high mobility [14,15,16]. In particular, metal halide variants with vacancy-ordered structures, such as Cs_2_SnCl_6_, Cs_2_HfCl_6_, and Cs_2_ZrCl_6_ [17,18,19], exhibit a unique 0D structure that facilitates electronic decoupling, thereby promoting the formation of self-trapped excitons (STEs) and resulting in stable emission [20,21]. However, achieving tunable emission bands and high photoluminescence quantum yield (PLQY) in A_2_BX_6_ metal halides remains challenging, limiting their application in various fields [22]. One effective approach to addressing this issue is doping with ns^2^ ions, such as Bi^3+^, Sb^3+^, and Te^4+^, to tailor the electronic and optical properties of metal halides [23]. The ns^2^ ions, characterized by their 5s^2^ outer electron configuration, cause significant lattice distortion upon incorporation into the metal halide lattice, which in turn induces the formation of photoinduced transient defects, leading to efficient radiative recombination of STEs [24,25]. Consequently, metal halides doped with ns^2^ ions typically exhibit broadband emission, high PLQY, large Stokes shifts, and wide full width at half maximum (FWHM), all of which are essential parameters for the fabrication of WLED devices. For example, Wang et al. successfully developed a wide range of tunable emission in Cs_2_ZrCl_6_ by co-doping with Bi^3+^ and Te^4+^. By varying the excitation wavelength, the emission could be tuned from blue to yellow light, and the resulting WLED device exhibited high-quality white light emission with a correlated color temperature (CCT) of approximately 5608 K and a color rendering index (CRI) of around 85.1 [26]. Wu’s research team co-doped Bi^3+^ and Sb^3+^ into Cs_2_SnCl_6_ to fabricate a WLED that emitted white light under excitation by a 380 nm UV LED chip. The device showed color coordinates of (0.30, 0.37), a CCT of 6800 K, and maintained approximately 68% of its photoluminescence intensity after 200 min of stability testing [27]. Despite these advances, current research has achieved relatively high CRI and good thermal stability for WLEDs. However, WLEDs based on dual-doped phosphors still face challenges under high-temperature operation. Differences in the thermal activation energies of the doped ions and the influence of electron–phonon interactions result in inconsistent changes in the luminescent properties of the two doped ions, leading to poor color stability. Additionally, the differences in diffusion rates and lattice matching between different ions during material synthesis can cause non-uniform doping. Therefore, the development of single-doped metal halide materials for WLED phosphors remains a critical challenge that needs to be addressed in this field.

In this paper, Sb^3+^-doped Rb_2_HfCl_6_ crystals were synthesized by a co-precipitation method. Upon Sb^3+^ doping, additional absorption peaks within the 260–400 nm range are introduced. The photoluminescence (PL) spectra reveal that the samples exhibit dual emission bands centered at 500 and 630 nm under 365 nm UV excitation. The photoluminescence excitation (PLE) spectra and time-resolved emission spectra indicate that the emission bands at 500 nm and 630 nm originate from the single- and triple-state STE radiative induced by the Jahn–Teller distortion of [SbCl_6_]^3−^ octahedron, respectively. Based on the dual broadband PL spectrum of Rb_2_HfCl_6_:Sb, its potential application in white light emitters is further explored. By combining Rb_2_HfCl_6_:12%Sb with a 365 nm LED chip, warm WLEDs were successfully fabricated, exhibiting broadband emission across the full visible wavelength range. The CIE chromaticity coordinates are (0.45, 0.41), the CCT is 3406 K, and the CRI reaches 81.9, demonstrating excellent color rendering performance. Moreover, the WLED exhibits outstanding stability under high driving currents, maintaining 95% of its emission intensity after 600 min of operation. In thermal stability tests, the PL intensity shows no significant attenuation after 20 heating–cooling cycles, and the emission intensity remains above 90% within the temperature range of 30 to 100 °C. Additionally, the luminescence intensity retains 90% of its initial value after 4 months of exposure to air. These results indicate that Rb_2_HfCl_6_:Sb holds significant potential in driving innovation and sustainable development in lighting technology, offering new opportunities and breakthroughs for the advancement of high-performance lighting technologies in the future.

## 2. Experimental Section

Materials: RbCl (99.95%) was purchased from Aladdin (Shanghai, China). HfCl_4_ (99.5%) and antimony chloride (SbCl_3_, 99%) were purchased from Shanghai Macklin Technology Co. (Shanghai, China) HCl (37 wt%) was purchased from Tianjin Chemical Reagent Factory (Tianjin, China).

Synthesis of Rb_2_Hf_1_Cl_6_:x%Sb^3+^ perovskite crystals: Firstly, 2 mmol RbCl were mixed with 3 mL HCl to form precursor solution A. x mmol SbCl_3_ and (1 − x) mmol HfCl_4_ were mixed with 8 mL of HCl, then heated to 45 °C and stirred for 0.5 h to produce precursor solution B. Afterward, solution A was dripped to solution B and continued to stir at 45 °C for one hour. The resulting suspension was then subjected to centrifugation at 9000 rpm for 10 min, and the precipitate was rinsed with ethanol four times. After drying at 60 °C for 6 h, the final product was acquired.

Characterizations: The crystal structures of the samples were analyzed through X-ray diffraction (Rigaku SmartLab SE, Rigaku, Tokyo, Japan) with Cu-Kα radiation (λ = 1.54178 Å). The scanning angle range is 10–80°, and the scanning speed is 5°/min. The morphology and elemental composition of the samples were analyzed by a ZEISS Gemini 300 scanning electron microscope (SEM) (ZEISS, Oberkochen, Germany) equipped with an energy dispersive spectrometer (EDS) attachment (OXFORD XPLORE30, Oxford Instruments, Abingdon, UK). X-ray photoelectron spectroscopy (XPS) analysis was performed using a Thermo Scientific K-Alpha spectrometer (Thermo Fisher Scientific, Waltham, MA, USA) equipped with a monochromatic Al Kα X-ray source (Thermo Fisher Scientific, Waltham, MA, USA) (hν = 1486.6 eV). The PLE, PL spectra, and temperature-dependent PL spectra were obtained using the Edinburgh spectrofluorometer FLS-1000 (Edinburgh Instruments Ltd., Livingston, UK) equipped with Xe 900 light (Edinburgh Instruments Ltd., Livingston, UK) as the excitation source. The fluorescence decay curves were collected by FLS-1000 spectrofluorometer with an external EPL laser (Edinburgh Instruments Ltd., Livingston, UK). The diffuse reflectance spectra were obtained by Shimadzu UV-3600i Plus (Shimadzu, Kyoto, Japan).

## 3. Results and Discussion

**Figure 1:**
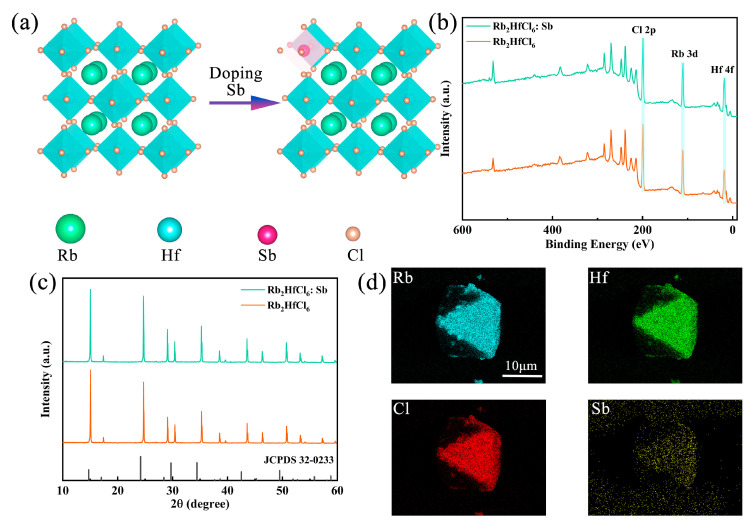
(**a**) Schematic crystal structures, (**b**) XPS spectra, and (**c**) XRD patterns of Rb_2_HfCl_6_ and Rb_2_HfCl_6_:Sb samples. (**d**) EDS element mapping of Rb, Hf, Cl, and Sb in Rb_2_HfCl_6_:Sb.

Sb^3+^-doped Rb_2_HfCl_6_ crystals were synthesized by the co-precipitation method using HfCl_4_, SbCl_3_, RbCl, and HCl as precursors. The crystal structures of Rb_2_HfCl_6_ and Sb^3+^-doped Rb_2_HfCl_6_ are illustrated in Figure 1a. The crystal system of Rb_2_HfCl_6_ is a cubic crystal system belonging to the Fm-3m space group, where each Hf atom is coordinated with six Cl atoms to form [HfCl6]^2−^ octahedra, and Rb atoms occupy the octahedral gaps, resulting in an ordered double perovskite structure. Upon Sb^3+^ doping, Sb atoms replace Hf atoms because of their similar coordination with Cl atoms, which reduces doping-induced strain and lattice defects. X-ray photoelectron spectroscopy (XPS) analysis is shown in Figure 1b, where characteristic peaks of Rb, Hf, and Cl are observed in both Rb_2_HfCl_6_ and Rb_2_HfCl_6_:Sb (note that the sample used for testing here is powder, and all subsequent tests will be powder). The high-resolution XPS spectrum in Figure S1 shows peaks at 538.69 and 531.84 eV belonging to Sb^3+^ 3d_3/2_ and 3d_5/2_, respectively. In addition, upon introducing Sb^3+^, the binding energies of Hf 4f (Figure S2) and Cl 2p (Figure S3) shift to higher energy, implying that Sb^3+^ was incorporated into the lattice of Rb_2_HfCl_6_ and occupied the Hf^4+^ site. X-ray diffraction (XRD) patterns of Rb_2_HfCl_6_ and Rb_2_HfCl_6_:Sb shown in Figure 1c match well with the standard card of Rb_2_HfCl_6_ (JCPDS #32-0233), confirming the phase purity. Notably, due to the larger ionic radius of Sb^3+^ (0.76 Å) compared to Hf^4+^ (0.71 Å), a noticeable shift in diffraction peaks to smaller angles occurs upon Sb^3+^ doping. The morphology and elemental composition of Rb_2_HfCl_6_:12%Sb^3+^ are exhibited in Figure 1d, the crystals are in the size range of 10–20 μm, and the mapping images of Rb, Hf, Sb, and Cl show uniform distributions of all elements, confirming the homogeneous incorporation of Sb^3+^ into the Rb_2_HfCl_6_ host lattice.

**Figure 2:**
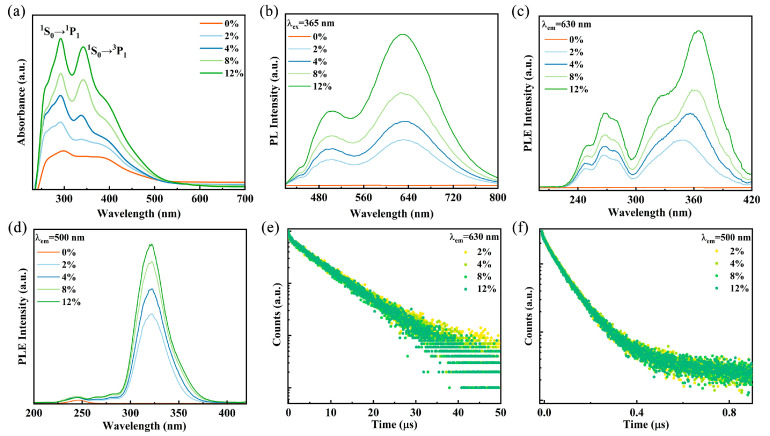
(**a**) Absorption spectra and (**b**) PL of Rb_2_HfCl_6_:x%Sb. PLE spectra of Rb_2_HfCl_6_:x%Sb monitored at (**c**) 630 nm and (**d**) 500 nm. PL decay curves of Rb_2_HfCl_6_:x%Sb monitored at (**e**) 500 nm and (**f**) 630 nm.

To investigate the optical properties of Sb^3+^-doped Rb_2_HfCl_6_, the reflectance spectra of the samples were characterized and converted to absorption spectra by the K-M equation. As shown in Figure 2a, the undoped Rb_2_HfCl_6_ exhibits uninterrupted absorption in 250–500 nm, which is consistent with previous reports [28]. After Sb^3+^ is introduced into Rb_2_HfCl_6_ crystals, the absorption spectra (Figure 2a) show additional absorption peaks. The peaks are located at 280–300 nm and 310–415 nm, which are attributed to the ^1^S_0_→^3^P_2_ and ^1^S_0_→^3^P_1_ transitions of Sb^3+^ ions, respectively [29,30].

It is well-established that ions with ns^2^ electronic configurations exhibit a ground state of ^1^S_0_, while their excited states are divided into four energy levels: the triplet states ^3^P_2_, ^3^P_1_, and ^3^P_0_, as well as the singlet state ^1^P_1_. According to transition principles and conversion rules, the transitions ^1^S_0_→^3^P_2_ and ^1^S_0_→^3^P_0_ are forbidden; however, lattice vibrations can facilitate these transitions. In contrast, the transitions ^1^S_0_→^1^P_1_ and ^1^S_0_→^3^P_1_ are allowed due to spin–orbit coupling [31,32]. The asymmetry in the doublet state arises from the dynamic Jahn–Teller effect, a common characteristic of ions with ns^2^ outer electron configurations, such as Sb^3+^, Te^4+^, and Bi^3+^ [24,32,33]. Under 245 nm excitation, Rb_2_HfCl_6_ exhibits a broad emission peak centered at 475 nm, with a large Stokes shift of 230 nm (Figure S4). This broad emission is attributed to the strong electron–phonon coupling in the perovskite structure with a soft lattice, leading to STE emission [34]. Upon Sb^3+^ doping, the PL spectra of Rb_2_HfCl_6_:x%Sb samples exhibit dual emission bands centered at 500 nm and 630 nm under 365 nm UV excitation, as shown in Figure 2b. The emission intensity increases with increasing Sb^3+^ concentration; however, the peak positions of the two emission bands remain nearly unchanged. Significantly, the emission spectra Rb_2_HfCl_6_:x%Sb cover a broad wavelength range of 400–800 nm, surpassing most previously reported wavelength ranges for Sb-doped compounds (Table S1). Furthermore, Gaussian peak deconvolution was performed on the emission spectrum of Rb_2_HfCl_6_:12%Sb. As shown in Figure S5. The spectrum reveals two distinct Gaussian components centered at 1.97 eV and 2.47 eV with full width at half maximum (FWHM) of 0.39 eV and 0.41eV, respectively. Figure 2c shows the PLE spectra of the Rb_2_HfCl_6_:x%Sb^3+^ sample monitored at 630 nm, the high-energy excitation band from 230 nm to 300 nm belongs to the lattice vibrationally assisted ^1^S_0_→^3^P_2_ transition, and the electrons of the ^3^P_2_ state jump to the ^3^P_1_ state through the Intersystem Crossing process [26]. The low-energy excitation band from 300 nm to 420 nm is assigned to the allowed spin–orbit coupling for the ^1^S_0_→^3^P_1_ transition [34,35]. In Figure 2d, the PLE spectra of the samples monitored at 500 nm show that the excitation band from 230 nm to 300 nm is attributed to the ^1^S_0_→^1^P_1_ transition, and the excitation band in the range of 300–400 nm is attributed to the ^1^S_0_→^3^P_1_ transition, which are similar to those monitored at 360 nm [36]. The PL decay curves of Rb_2_HfCl_6_:x%Sb monitored at 500 nm and 630 nm are shown in Figure 2e,f. The curves show a clear second-order decay process, which can be fitted by a double-exponential decay function, as follows [37]:

(1) $$I\left(t\right)=A_{1}\exp\left(-\frac{t}{\tau_{1}}\right)+A_{2}\exp\left(-\frac{t}{\tau_{2}}\right)$$

where, *τ*_1_ and *τ*_2_ denote the lifetimes of the different processes. *A*_1_ and *A*_2_ represent their relative contributions to the luminescence intensity. The fitting results are shown in Tables S2 and S3; the lifetimes are essentially unchanged as the Sb^3+^ doping concentration varies. In addition, the lifetimes at the two emission centers are different, suggesting that the two luminescence centers stem from two excited state energy levels [38].

**Figure 3:**
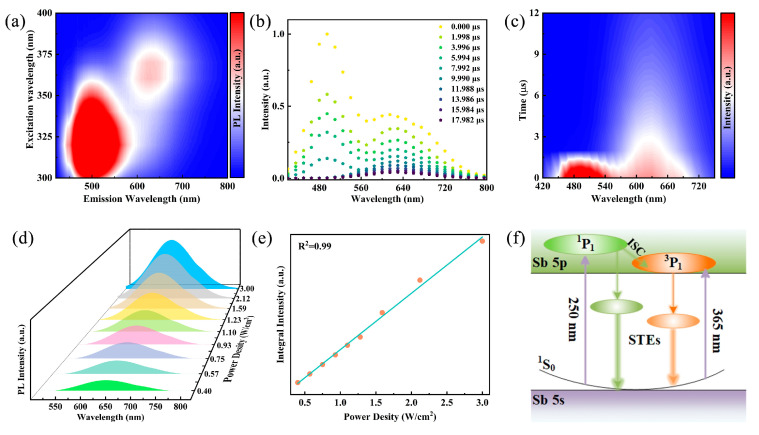
(**a**) Pseudo color map of excitation wavelength-dependent photoluminescence of Rb_2_HfCl_6_:Sb. (**b**) Time-resolved emission spectra of Rb_2_HfCl_6_:Sb under 340 nm excitation. (**c**) Pseudo color map of decay mapping of Rb_2_HfCl_6_:Sb versus emission wavelength under 340 nm excitation. (**d**) PL spectra of Rb_2_HfCl_6_:Sb samples under varying excitation power densities and (**e**) relationship of corresponding emission intensity and excitation power density. (**f**) Schematic diagram of luminescence mechanism of Rb_2_HfCl_6_:Sb.

To further elucidate the luminescence mechanism, excitation wavelength-dependent emission spectra of Rb_2_HfCl_6_:12%Sb were performed. As shown in Figure 3a, the PL pseudo color map of the sample under excitation wavelengths ranging from 300 to 390 nm (with 10 nm intervals) reveals that the luminescence centers correspond to approximately 500 nm and 630 nm within the high- and low-energy excitation wavelength ranges, respectively. Typically, ionic emission depends on energy transfer related to the excitation wavelength. However, the normalized PL spectra of Rb_2_HfCl_6_:12%Sb in Figure S6a demonstrate that the central positions and shapes of the two emission peaks at 500 nm and 630 nm remain unchanged with varying excitation wavelengths, which rules out the possibility of ionic emission [28]. Furthermore, the normalized PLE spectra of Rb_2_HfCl_6_:12%Sb monitored at different emission wavelengths (Figure S6b) exhibit four excitation peaks. The two high-energy excitation peaks correspond to the ^1^S_0_→^1^P_1_ and ^1^S_0_→^3^P_2_ transitions, respectively, while the two low-energy excitation peaks originate from the ^1^S_0_→^3^P_1_ transition induced by Jahn–Teller splitting [39]. Additionally, the shapes of each excitation band remain unchanged, confirming that the two broad emissions at 500 nm and 630 nm arise from the relaxation of excited states rather than surface traps or lattice defects. In addition, the time-resolved emission spectra of Rb_2_HfCl_6_:12%Sb under 365 nm excitation were conducted. As shown in Figure 3b, a rapid decrease in intensity centered at 500 nm and a slow decrease in intensity centered at 630 nm are observed over the duration of the excitation pulse, suggesting that the emission peak centered at 630 nm decays more slowly [36]. The time-resolved pseudo color maps under 365 nm excitation in Figure 3c show different emission centers at 500 nm and 630 nm. Therefore, we infer that the STE emission at 500 nm and 630 nm originates from the singlet and triplet states within the [SbCl6]^3−^ octahedron, respectively [29]. It is noteworthy that the permanent defect emission typically exhibits broad emission bands, and the photoluminescence intensity saturates at higher excitation power densities. The power-dependent PL spectra of Rb_2_HfCl_6_:Sb shown in Figure 3d reveal a steady increase in emission intensity with increasing excitation power densities, maintaining a consistent emission peak shape. Thus, the possibility of permanent defective emission was ruled out by the observed linear correlation between luminescence intensity and power density (Figure 3e) [40]. The above results indicate that the luminescence of Rb_2_HfCl_6_:Sb originates from STE emission caused by the Jahn–Teller distortion of the [SbCl6]^3−^ octahedron, which is characterized by broadband emission with almost constant wavelength-dependent spectra and a lifetime of microseconds [34,38,41]. In summary, the luminescence mechanism of Rb_2_HfCl_6_:Sb is proposed, as shown in Figure 3f. Under high-energy UV irradiation, the electrons in the ^1^S_0_ state were excited to the ^1^P_1_ state, and then some of the electrons jumped from the ^1^P_1_ state to the ^3^P_1_ state through an intersystem cross-relaxation process. Under low-energy UV irradiation, electrons on the ^1^S_0_ state are excited to the ^3^P_1_ state. Afterward, the electrons located in the ^1^P_1_ and ^3^P_1_ states are transferred to the single and triple self-trapped states with lower intrinsic energies and finally radiative transition back to the ^1^S_0_ state to achieve cyan (500 nm) and orange-red (630 nm) dual broadband emission.

**Figure 4:**
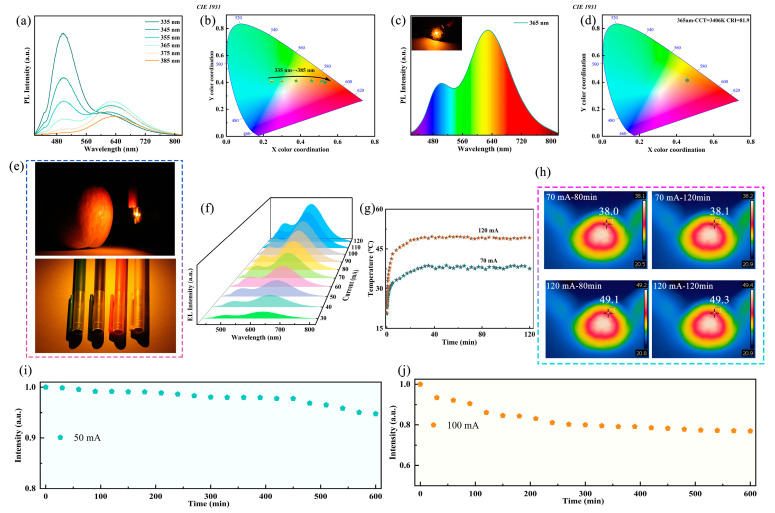
(**a**) PL spectra and (**b**) corresponding CIE color coordinates of Rb_2_HfCl_6_:12%Sb samples under 335–385 nm excitation. (**c**) PL spectra of WLED based on Rb_2_HfCl_6_:12%Sb phosphor (inset: photograph of working WLED). (**d**) CIE color coordinates of WLED driving by 365 nm chip. (**e**) Photos of apple and crayons under WLED lighting. (**f**) EL spectra of WLED at different driving currents. (**g**) Temperature changes in WLED with running time under driving currents of 70 and 120 mA, respectively. (**h**) Thermography photos of WLED at driving current of 70 mA and 120 mA. EL intensity of WLEDs running at current of (**i**) 50 mA and (**j**) 100 mA for 600 min.

The dual broadband emission characteristics of Sb-doped Rb_2_HfCl_6_ exhibit great potential as a white light emitter. To confirm the potential application of Rb_2_HfCl_6_:Sb in WLEDs, the excitation wavelength-dependent PL spectra of Rb_2_HfCl_6_:12%Sb were measured. In Figure 4a, as the excitation wavelength varies from 335 nm to 385 nm, the emission intensity ratio of 500 nm and 630 nm gradually decreases. This corresponds to a shift in emission color from the cyan region to the orange-red region, with the CIE coordinates adjusting from (0.246, 0.411) to (0.533, 0.399) (Figure 4b). To further confirm the potential application of Rb_2_HfCl_6_:12%Sb in single-component solid-state lighting, a WLED was constructed through the integration of phosphor with a commercial 365 nm LED chip. Figure 4c shows that the working WLED emits bright warm white light, exhibiting broadband radiation across the complete visible spectrum. The corresponding CIE coordinates are (0.45, 0.41), located in the warm white light region with a CCT of 3406 K (Figure 4d). Notably, the WLED exhibits a CRI of 81.9, significantly higher than that of conventional fluorescent lamps (≈65) [12]. This single-component WLED avoids the common issues associated with mixed phosphors, such as low luminescence efficiency, insufficient color rendering, and complex fabrication processes. Furthermore, compared to co-doped metal halide phosphors, single-doped ones generally exhibit better color stability. As illustrated in Figure 4e, the fabricated WLED exhibits remarkable color resolution and visual performance, where apples and crayons appear vivid and realistic under its illumination. To further verify the potential application of Rb_2_HfCl_6_:12%Sb in solid-state lighting, electroluminescence (EL) spectra were collected under different driving currents, as shown in Figure 4f. As the driving current increases, the EL intensity gradually enhances (Figure S7), and the CIE coordinates remain unchanged (Figure S8), indicating the luminescence stability of Rb_2_HfCl_6_:Sb. Figure 4g presents the WLED surface temperature at driving currents of 70 and 120 mA, showing a rapid increase in the first 20 min before reaching thermal equilibrium. During an extended operation of 120 min, the monitored surface temperature remains stable. As shown in Figure 4h, an infrared camera was used to monitor the surface temperature of the WLED at different driving currents over time. Under continuous excitation at 70 and 120 mA for 80 and 120 min, the surface temperature remains stable, indicating that the device can maintain a consistent temperature even under high driving currents. To further investigate the working stability of the fabricated WLED, continuous emission intensity measurements were conducted at constant driving currents of 50 and 100 mA. As shown in Figure 4i, after 600 min of operation at 50 mA, the WLED retains 95% of its initial emission intensity. Moreover, at a higher driving current of 100 mA (Figure 4j), the emission intensity remains above 80%. These results demonstrate that the fabricated WLED exhibits excellent stability even under high current operation.

**Figure 5:**
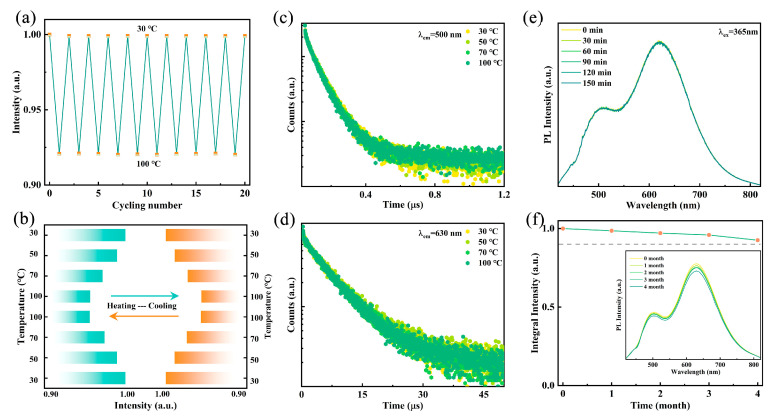
(**a**) Switching of emission intensities of Rb_2_HfCl_6_:12%Sb under 365 nm excitation with cooling and heating cycles between 30 °C and 100 °C. (**b**) Temperature-dependent emission intensities of sample in first and last cycles. Luminescence decay curves monitored at (**c**) 500 nm and (**d**) 630 nm under different temperatures. (**e**) PL spectra of Rb_2_HfCl_6_:12%Sb with different times at 60 °C under excitation of 365 nm. (**f**) PL spectra and integrated intensities of samples stored in air for different times.

For WLED device applications, the luminescence stability of the emitter against heat, ambient oxygen, and moisture is crucial. As shown in Figure 5a, after 20 heating–cooling cycles, the PL intensity exhibits no significant degradation, demonstrating stability superior to that of conventional lead-based halides [42]. As illustrated in Figure 5b, the PL intensity of the sample at 100 °C remains above 90% of its intensity at 30 °C. To further verify its thermal stability, temperature-dependent fluorescence decay curves at 500 nm and 630 nm were measured. As illustrated in Figure 5c,d, the fluorescence lifetimes remain nearly unchanged when heated from 30 °C to 100 °C, indicating excellent thermal resistance, which surpasses previously reported metal halide WLED devices. Figure 5e shows that after continuous heating at 60 °C for 150 min, both the spectral shape and emission intensity remain stable, further confirming the high thermal stability of Rb_2_HfCl_6_:12%Sb. Additionally, after four months of exposure to air, the emission intensity retains 90% of its initial value, indicating high resistance to oxygen and moisture degradation (Figure 5f). These results suggest that Rb_2_HfCl_6_:12%Sb exhibits remarkable stability, making it suitable for practical applications in harsh environments. Owing to its environmentally benign characteristics, superior color rendering properties, and facile fabrication process, Rb_2_HfCl_6_:12%Sb emerges as an excellent luminescent material for advanced WLED applications.

## 4. Conclusions

In summary, Sb^3+^-doped Rb_2_HfCl_6_ perovskite crystals were successfully synthesized via a co-precipitation method. XRD and XPS analyses confirm that Sb^3+^ is incorporated into the Rb_2_HfCl_6_ lattice, substituting Hf^4+^ sites. Optical and luminescence characterizations reveal that the dual-emission bands originate from the Jahn–Teller distortion-induced singlet and triplet STE radiative transitions within the [SbCl_6_]^3−^ octahedra. The excitation wavelength-dependent emission properties enable tunable luminescence from cyan to orange-red by adjusting the excitation wavelength, making it an ideal material for single-component WLEDs. Thus, the WLED fabricated using Rb_2_HfCl_6_:12%Sb exhibits excellent performance, with a high CRI of 81.9 and outstanding operational stability. Notably, this material demonstrates exceptional thermal stability, retaining over 90% of its emission intensity at 100 °C and showing no significant degradation after 20 heating–cooling cycles. Additionally, after four months of exposure to air, the emission intensity remains at 90% of its initial value, indicating strong resistance to humidity and oxygen. Compared to lead-based perovskites, mixed phosphors, and co-doped phosphors, Rb_2_HfCl_6_:Sb offers advantages such as environmental friendliness, simple fabrication, and stable performance, making it a promising emitter for WLEDs. This work provides new insights into the development of next-generation high-temperature-resistant and highly reliable optoelectronic devices while expanding their potential applications in extreme environments and intelligent sensing.

## Data Availability

The original contributions presented in this study are included in the article/Supplementary Materials. Further inquiries can be directed to the corresponding author.

## Supplementary Materials

The following supporting information can be downloaded at: https://www.mdpi.com/article/10.3390/ma18091896/s1, Figure S1: High-resolution XPS spectra of Sb 3d in Rb_2_HfCl_6_:Sb; Figure S2: High-resolution XPS spectra of Cl 2p in Rb_2_HfCl_6_ and Rb_2_HfCl_6_:Sb; Figure S3: High-resolution XPS spectra of Hf 4f in Rb_2_HfCl_6_ and Rb_2_HfCl_6_:Sb; Figure S4: PL and PLE spectra of Rb_2_HfCl_6_; Figure S5: Gaussian peak fitting for emission spectra of Rb_2_HfCl_6_:12%Sb; Figure S6: (a) Normalized PL spectra of Rb_2_HfCl_6_:12%Sb under excitation of 300–390 nm. (b) Normalized PLE spectra of Rb_2_HfCl_6_:12%Sb monitored at 480–680 nm; Figure S7: Integrated EL intensity of WLED pumped by 365 nm LED chip under different driving currents; Figure S8: CIE chromaticity coordinates of electroluminescence spectra for red LEDs under different currents; Table S1: Comparison of emission spectra range of different Sb^3+^-doped compounds [43,44,45,46,47]; Table S2: Fitted PL lifetime of Rb_2_HfCl_6_:x%Sb monitored at 500 nm; Table S3: Fitted PL lifetime of Rb_2_HfCl_6_:x%Sb monitored at 630 nm.
