# Supplementary material for: Sb3+-Doped Rb2HfCl6 Perovskites as High-Performance Thermally Stable Single-Component Phosphors for White Light-Emitting Diodes
Source: Materials (Basel). 2025 Apr 22;18(9):1896. doi: 10.3390/ma18091896 (PMC12072897; doi:10.3390/ma18091896)
Supplement: Supplementary file 1 [file materials-18-01896-s001.zip › materials-3568648-supplementary.pdf]

Sb^{3+}-Doped Rb_2HfCl_6 Perovskites as High-Performance Thermally Stable Single-Component Phosphors for White Light-Emitting Diodes

Yanbiao Li and Yuefeng Gao *

College of Marine Engineering, Dalian Maritime University, Dalian 116026, China; dmulyb@dlmu.edu.cn
* Correspondence: yue232feng@163.com; Tel.: +86-0411-84729314

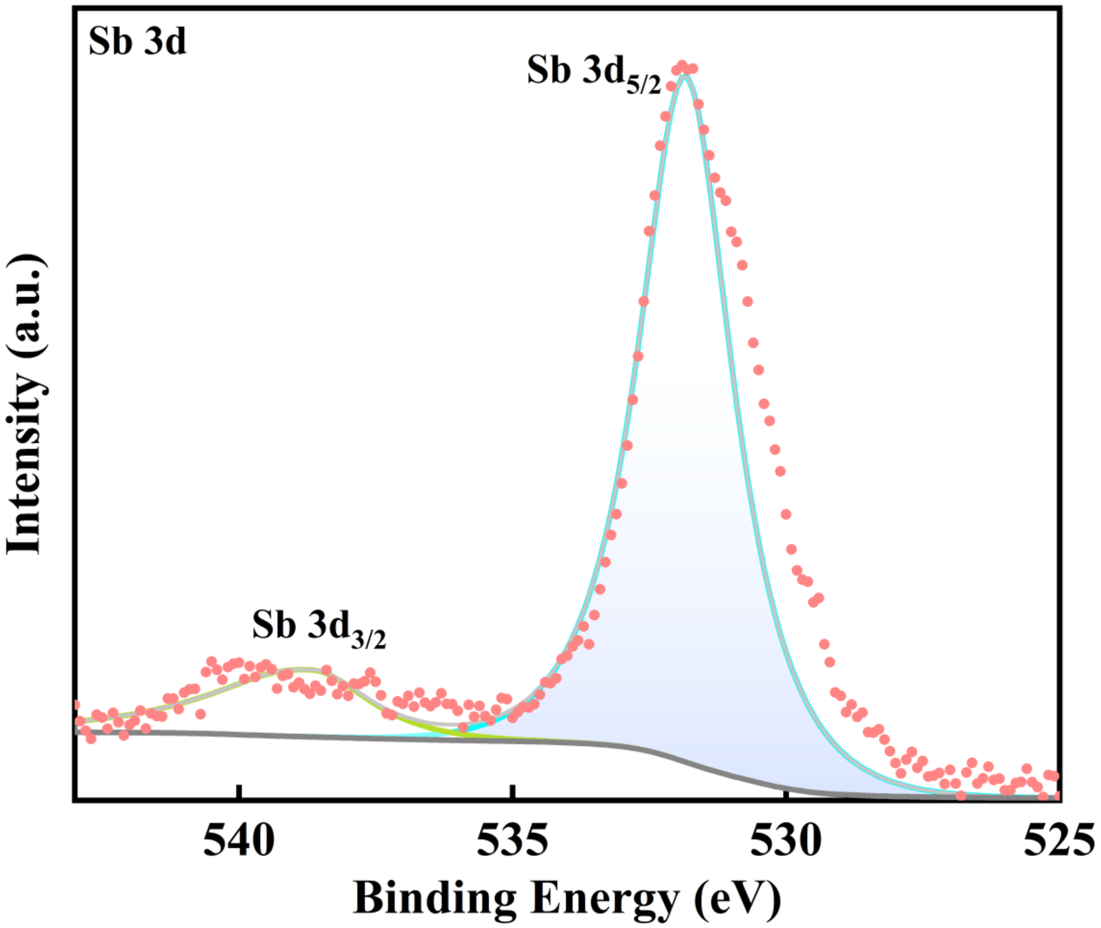

Figure S1. High-resolution XPS spectrum of Sb 3d in Rb_2HfCl_6: Sb.

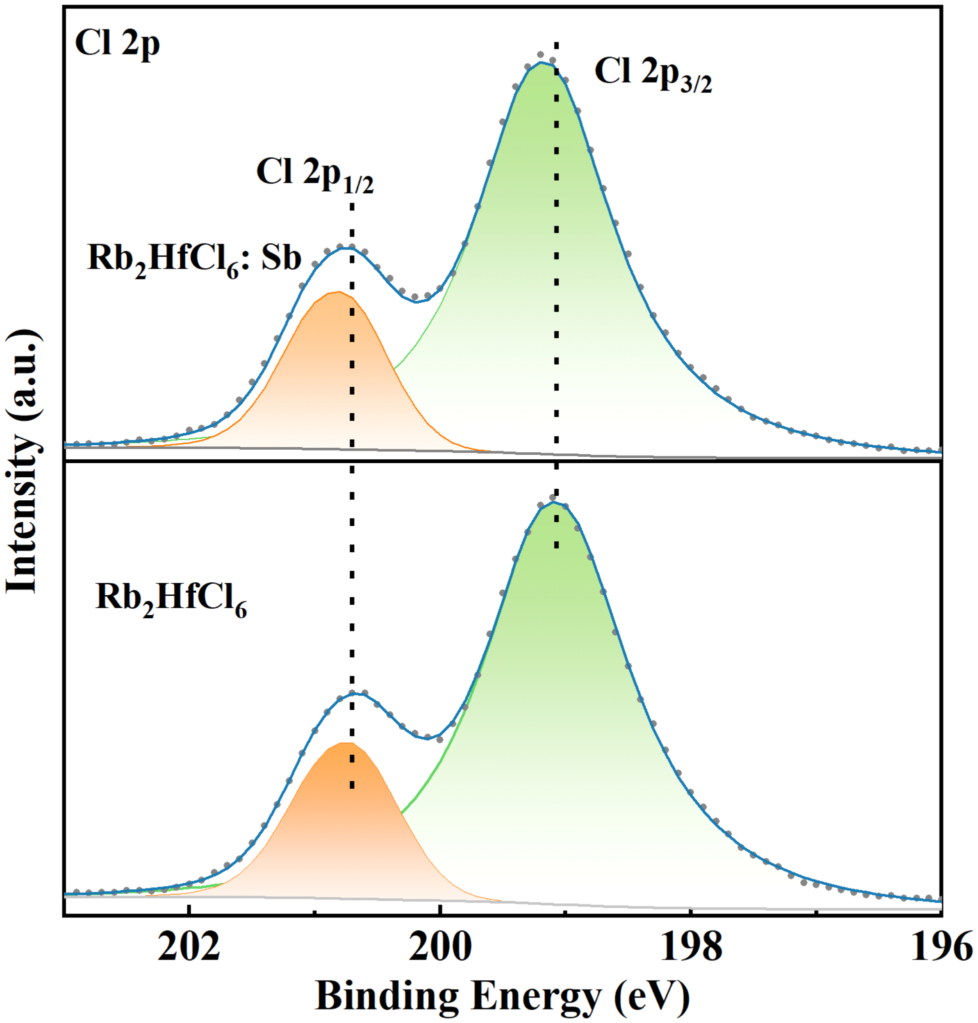

Figure S2. High-resolution XPS spectra of Cl 2p in Rb_2HfCl_6 and Rb_2HfCl_6: Sb.

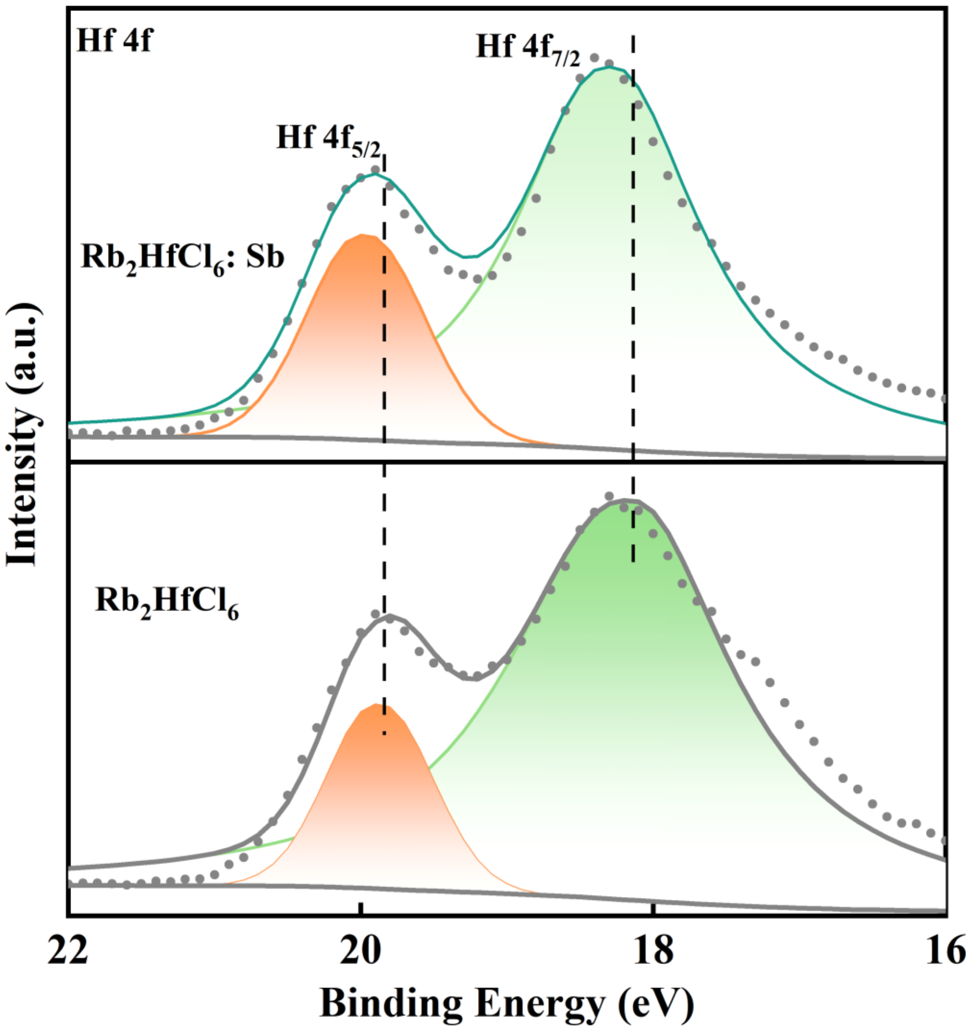

Figure S3. High-resolution XPS spectra of Hf 4f in Rb_2HfCl_6 and Rb_2HfCl_6: Sb.

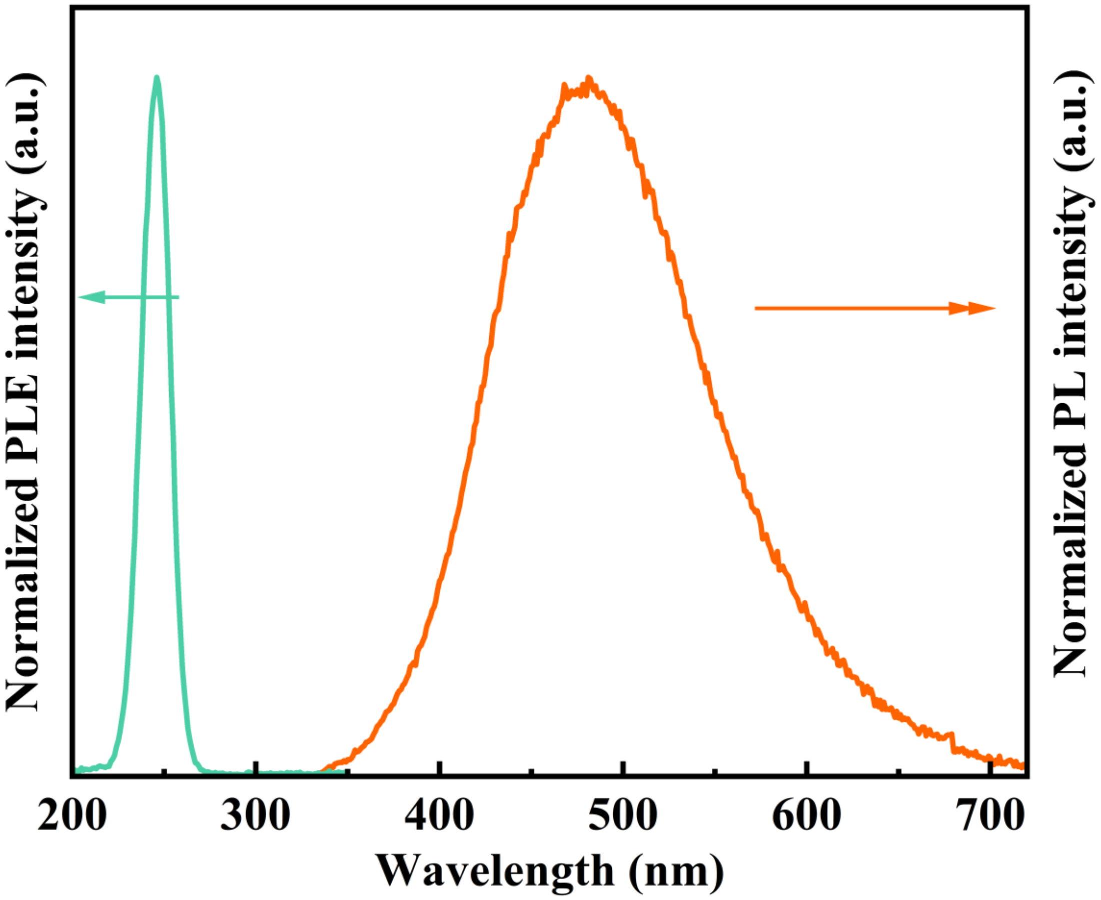

Figure S4. PL and PLE spectra of Rb_2HfCl_6.

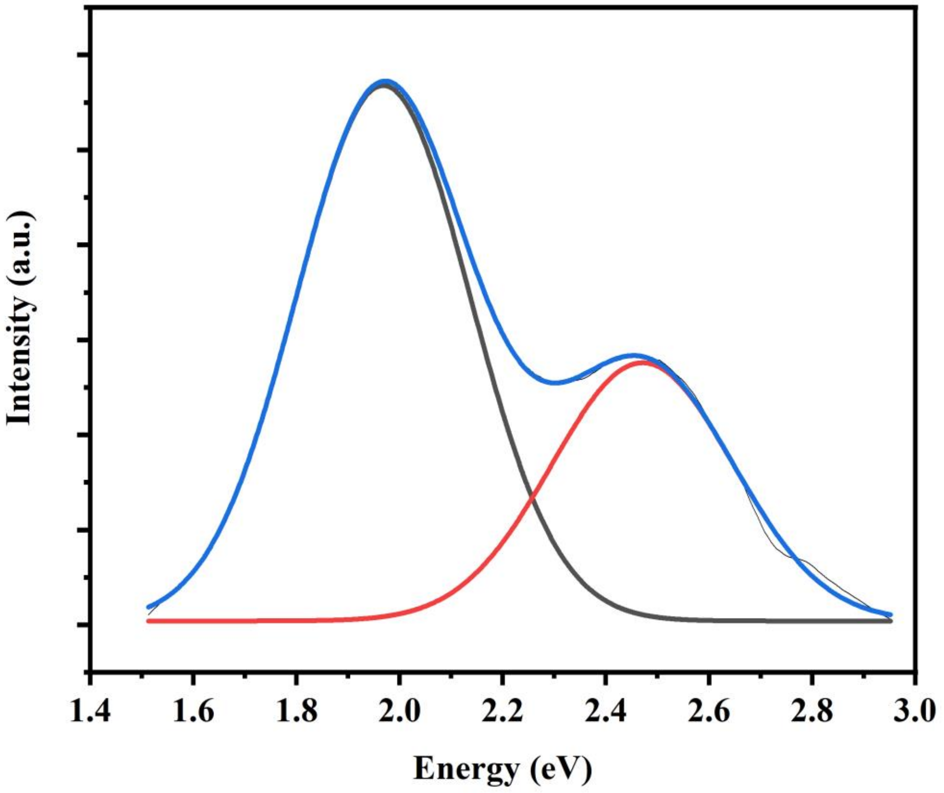

Figure S5. Gaussian peak fitting for the emission spectrum of Rb_2HfCl_6: 12%Sb.

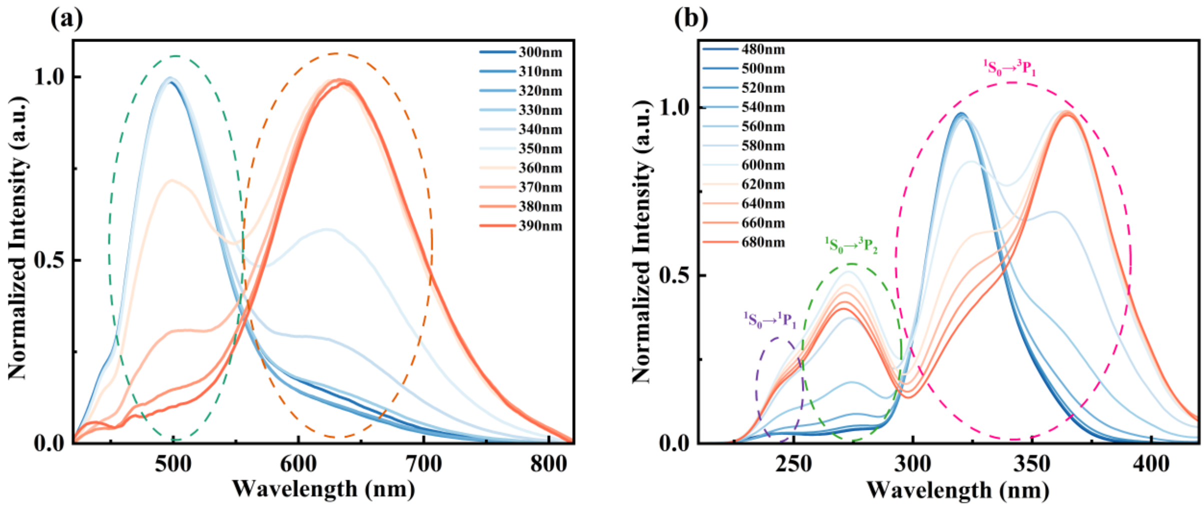

Figure S6. a) Normalized PL spectra of Rb_2HfCl_6: 12%Sb under excitation of 300-390 nm. b) Normalized PLE spectra of Rb_2HfCl_6: 12%Sb monitored at 480-680nm.

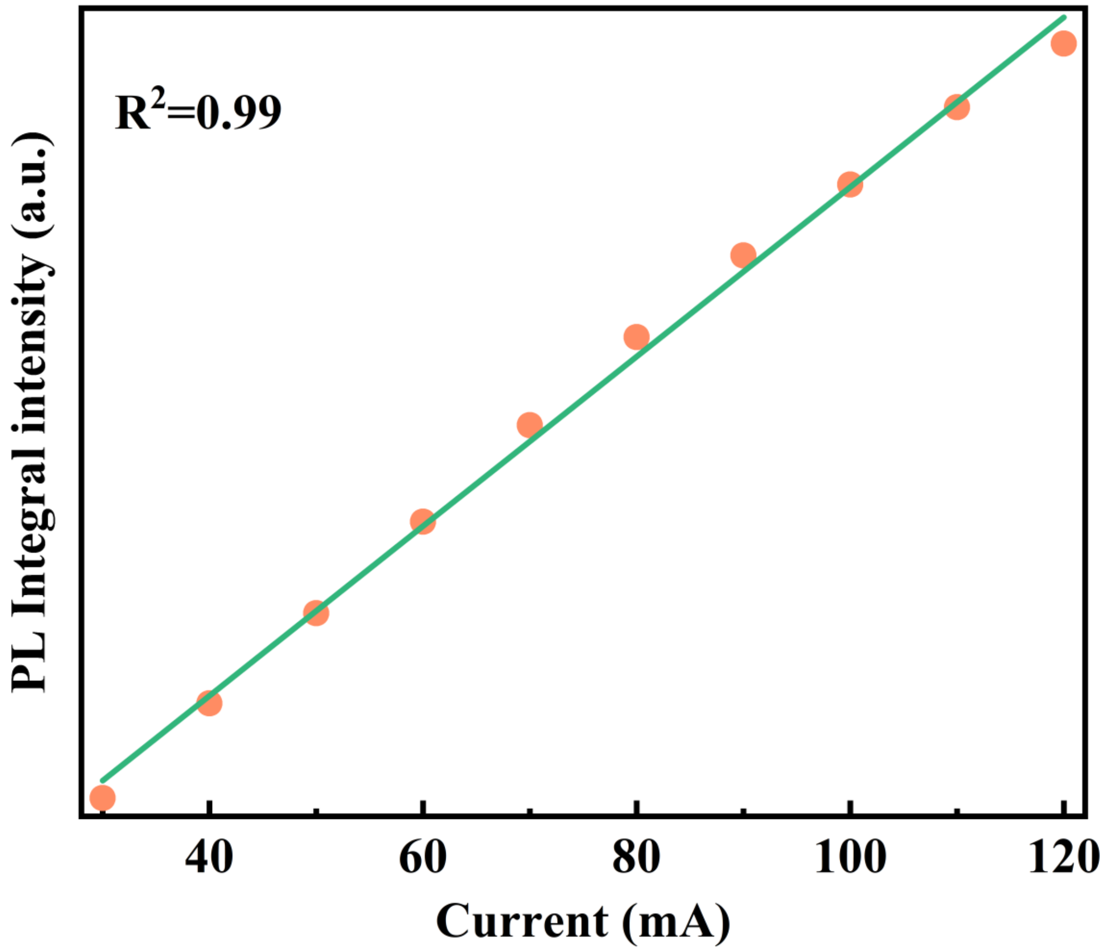

Figure S7. The integrated EL intensity of WLED pumped by a 365 nm LED chip under different driving currents.

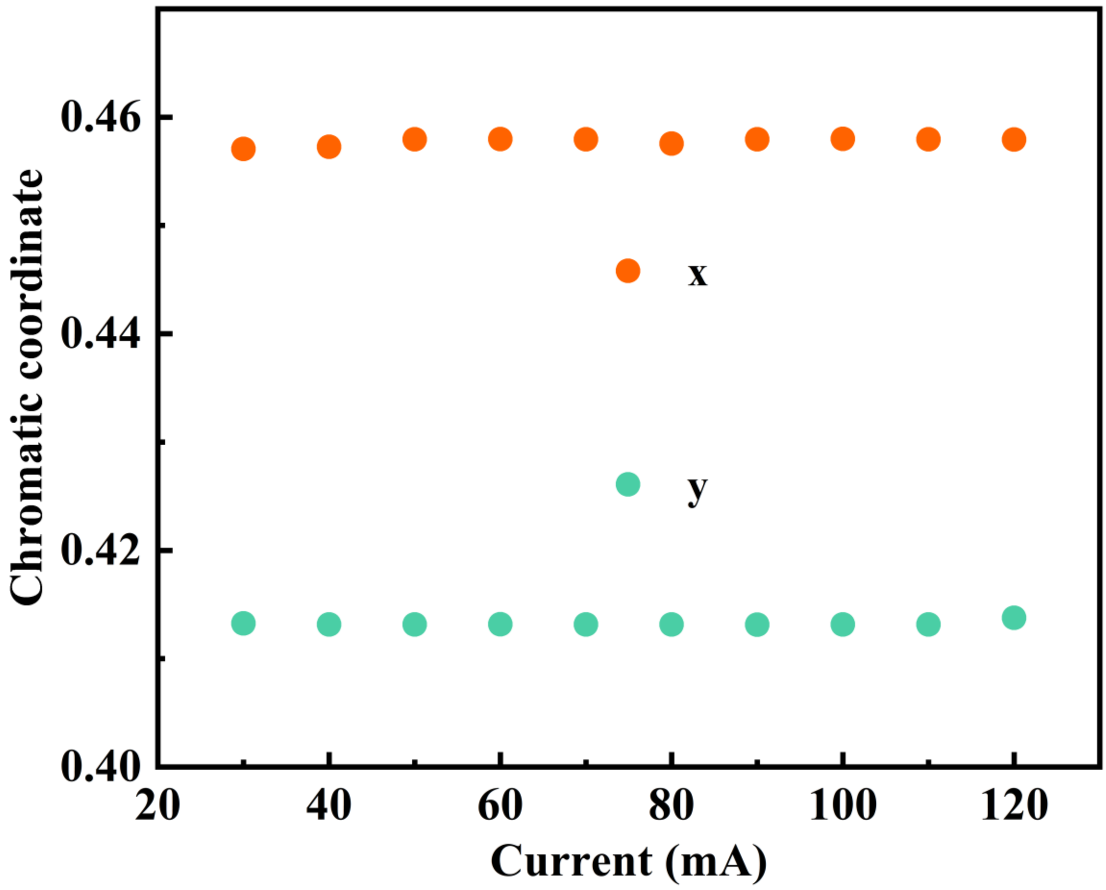

Figure S8. CIE chromaticity coordinates of electroluminescence spectra for red LEDs under different currents.

Table S1. Comparison of emission spectra range of different Sb^{3+} doped compounds.

Material	Emission range (nm)	Ref.
$Cs_2NaInCl_6$: Sb^{3+}	370-550	[1]
Cs_2SnCl_6: Sb^{3+}	500-700	[2]
$Cs_3Cd_2Cl_7$: Sb^{3+}	480-600	[3]
$Cs_4Cd_2Cl_6$: Sb^{3+}	425-575	[4]
Cs_2ZnCl_4: Sb^{3+}	600-1000	[5]
Rb_2HfCl_6: Sb^{3+}	420-800	This Work

Table S2. Fitted PL lifetime of Rb_2HfCl_6:x%Sb monitored at 500 nm.

x	A_1 (%)	τ_1 (ns)	A_2 (%)	τ_2 (ns)	τ_{ave} (ns)
2	15.3	19.24	84.7	85.34	82.77
4	19.9	26.40	80.1	89.30	85.00
8	20.7	25.03	79.3	87.89	83.53
12	16.0	20.22	84.0	85.51	82.69

Table S3. Fitted PL lifetime of Rb_2HfCl_6:x%Sb monitored at 630 nm.

x	A_1 (%)	τ_1 (ns)	A_2 (%)	τ_2 (ns)	τ_{ave} (ns)
2	26.4	0.26	73.6	7.12	7.01
4	27.3	0.25	62.7	7.23	7.06
8	28.1	0.28	71.9	7.15	7.00
12	24.7	0.31	75.3	7.01	6.93

References

1. Liu, X.; Xu, X.; Li, B.; Yang, L.; Li, Q.; Jiang, H.; Xu, D. Tunable Dual-Emission in Monodispersed Sb^{3+}/Mn^{2+} Codoped $Cs_2NaInCl_6$ Perovskite Nanocrystals through an Energy Transfer Process. Small 2020, 16, 2002547.
2. Li, J.; Tan, Z.; Hu, M.; Chen, C.; Luo, J.; Li, S.; Gao, L.; Xiao, Z.; Niu, G.; Tang, J. Antimony Doped Cs_2SnCl_6 with Bright and Stable Emission. Front. Optoelectron. 2019, 12, 352–364.
3. Dai, G.; Ma, Z.; Qiu, Y.; Ma, Z. Codoped 2D All-Inorganic Halide Perovskite $Cs_3Cd_2Cl_7$:Sb^{3+} :Mn^{2+} with Ultralong Afterglow. Inorg. Chem. 2023, 62, 7906–7913.
4. Huang, D.; Zheng, P.; Cheng, Z.; Ouyang, Q.; Lian, H.; Lin, J. Metal Halides $RbCdCl_3$:Sb^{3+} and Rb_4CdCl_6:Sb^{3+} with Yellow and Cyan Emissions Obtained via a Facile Hydrothermal Process. J. Mater. Chem. C 2023, 11, 16390–16397.
5. Zhang, W.; Chen, G.; Lu, X.; Wang, Y.; Zhang, N.; Zhang, Q.; Liu, X.; Tang, X. Unveiling Sb^{3+} Doping and Tricolor Luminescence from Intrinsic Self-Trapped Excitons in Cs_2ZnCl_4 Crystals. J. Phys. Chem. Lett. 2024, 15, 2616–2623.
